# Dr. P. W. Johns, Hot Springs

**Published:** 1892-09

**Authors:** 


DR. P. W. JOHNS.—We are in receipt of a letter from Dr. E. P. Becton, of Sulphur Springs, Texas, from which we extract the following, believing it will be read with interest by Dr. Johns' numerous friends in Texas:

"While there (Hot Springs) I had the pleasure of spending some time with our friend, Dr. P. W. Johns. He is in fine health and is recognized there as he always was here as a cultured gentleman and an ethical physician. I am sorry I cannot say as much for all who are practicing in that city. Dr. Johns is living up to every requirement of the code and professionally is making a success which is truly gratifying to his friends."

Dr. Johns, whom we knew in "Knickerbockers" thirty years ago, comes of the right kind of stock to make a success in anything that is honorable and high principled. We congratulate *Hot Springs*.

